# Structural plasticity of GABAergic axons is regulated by network activity and GABA_A_ receptor activation

**DOI:** 10.3389/fncir.2013.00113

**Published:** 2013-06-26

**Authors:** Anne Schuemann, Agnieszka Klawiter, Tobias Bonhoeffer, Corette J. Wierenga

**Affiliations:** Department Synapses, Plasticity, Circuits, Max Planck Institute of NeurobiologyMartinsried, Germany

**Keywords:** activity-dependent plasticity, bouton dynamics, hippocampal organotypic cultures, inhibitory axons, two-photon microscopy

## Abstract

Coordinated changes at excitatory and inhibitory synapses are essential for normal brain development and function. It is well established that excitatory neurons undergo structural changes, but our knowledge about inhibitory structural plasticity is rather scarce. Here we present a quantitative analysis of the dynamics of GABAergic boutons in the dendritic region of the hippocampal CA1 area using time-lapse two-photon imaging in organotypic hippocampal cultures from GAD65-GFP mice. We show that ~20% of inhibitory boutons are not stable. They are appearing, disappearing and reappearing at specific locations along the inhibitory axon and reflect immature or incomplete synapses. Furthermore, we observed that persistent boutons show large volume fluctuations over several hours, suggesting that presynaptic content of inhibitory synapses is not constant. Our data show that inhibitory boutons are highly dynamic structures and suggest that inhibitory axons are continuously probing potential locations for inhibitory synapse formation by redistributing presynaptic material along the axon. In addition, we found that neuronal activity affects the exploratory dynamics of inhibitory axons. Blocking network activity rapidly reduces the number of transient boutons, whereas enhancing activity reduces the number of persistent inhibitory boutons, possibly reflecting enhanced competition between boutons along the axon. The latter effect requires signaling through GABA_A_ receptors. We propose that activity-dependent regulation of bouton dynamics contributes to inhibitory synaptic plasticity.

## Introduction

In a healthy brain, the balance between excitatory and inhibitory synapses is dynamically regulated and changes in excitatory and inhibitory synapses often occur in a coordinated fashion (Liu, 2004; Bourne and Harris, 2011; Chen et al., 2012). Local synaptic imbalances are thought to underlie neurodevelopmental diseases such as autism or schizophrenia (Rubenstein and Merzenich, 2003; Yizhar et al., 2011; Grillo et al., 2012). It is well established that functional adaptations of neuronal networks are often reflected in structural changes of the neurons involved (Bailey and Kandel, 1993; Yuste and Bonhoeffer, 2001; Holtmaat and Svoboda, 2009). For excitatory synapses these changes often occur on the level of dendritic spines, and these have been studied extensively (Engert and Bonhoeffer, 1999; Trachtenberg et al., 2002; Holtmaat et al., 2005; Keck et al., 2008; Hofer et al., 2009; Yang et al., 2009). However, structural changes of inhibitory neurons have also been reported, in axons (Keck et al., 2008; Marik et al., 2010; van Versendaal et al., 2012) as well as in dendrites (Keck et al., 2011; Chen et al., 2012). Inhibitory changes often precede and possibly facilitate excitatory changes during adult plasticity (Rosier et al., 1995; Hensch, 2005; Froemke et al., 2007; Keck et al., 2011; Chen et al., 2012; van Versendaal et al., 2012). Yet, despite the importance of structural plasticity of inhibition, our current knowledge of inhibitory structural dynamics is rather scarce.

The majority of inhibitory synapses are located on dendrites of principal cells (Megías et al., 2001) and dendritic inhibition plays an important role in the integration of synaptic signals (Murayama et al., 2009; Gidon and Segev, 2012; Lovett-Barron et al., 2012; Müller et al., 2012). While glutamatergic synapses are typically formed on dendritic spines, GABAergic synapses are usually formed directly on the dendritic shaft (Gottlieb and Cowan, 1972; Harris and Kater, 1994). The presynaptic structures of both types of synapses are formed by axonal varicosities (boutons) containing transmitter-filled synaptic vesicles. For excitatory synapses, it has been shown that presynaptic boutons exchange material, including synaptic vesicles and components of the release machinery, via the axonal shaft (Krueger et al., 2003; Darcy et al., 2006; Sabo et al., 2006; McAllister, 2007; Staras et al., 2010). The continuous exchange of material makes boutons highly dynamic structures, allowing for rapid formation of new boutons, but at the same time renders presynaptic boutons vulnerable to competition with neighboring boutons on the same axon. In contrast to excitatory synapses which are often formed through the extension of new dendritic protrusions, the formation of inhibitory synapses is not mediated by protrusions. Instead, new inhibitory boutons emerge *de novo* at locations where the inhibitory axon is already in close contact to a dendrite (Wierenga et al., 2008). This suggests that presynaptic bouton dynamics are especially important for the formation and plasticity of dendritic inhibitory synapses.

With a dynamic regulation of the balance between excitation and inhibition it is expected that inhibitory synapses adapt to changes in excitatory activity. Here we examine how structural changes of inhibitory axons are regulated by activity. We investigate in detail the dynamics of presynaptic GABAergic boutons in hippocampal organotypic cultures in control conditions and when activity levels were enhanced or reduced. Using time-lapse two-photon microscopy followed by immunohistochemistry we could distinguish between persistent GABAergic boutons, presumably reflecting established inhibitory synapses, and a population of non-persistent boutons, including incomplete or immature synapses in the process of formation or disassembly. Furthermore, we show how the dynamics of GABAergic boutons are rapidly affected by changes in network activity, using quantitative analysis of bouton turnover and volume changes. Finally, we provide evidence for the involvement of GABA_A_ receptor activation in these processes.

## Materials and methods

### Slice cultures and pharmacological treatments

Hippocampal slices (350 μm thick) were prepared from postnatal day 4–6 GAD65-GFP mice (López-Bendito et al., 2004), and maintained in a roller incubator at 35°C (Gähwiler, 1981). Slices were kept in culture for at least 10 days before the experiments [range: 10–17 days *in vitro* (DIV)]. At this maturation stage, axonal densities were stable (data not shown). In GAD65-GFP mice, ~20% of all hippocampal CA1 interneurons express GFP (Wierenga et al., 2010) and the expression level is stable from early embryonic age into adulthood (López-Bendito et al., 2004). GFP-expressing interneurons are mostly dendritically targeting, VIP and reelin-positive, interneurons (Wierenga et al., 2010). The low abundance of GFP-expressing axons is crucial for the analysis of boutons along individual axons.

For the experiments, slices were transferred to a recording chamber, where they were continuously perfused with carbogenated (95% O_2_, 5% CO_2_) artificial cerebrospinal fluid (ACSF; containing 126 mMNaCl, 3 mMKCl, 2.5 mM CaCl_2_, 1.3 mM MgCl_2_, 1.25 mM NaPH_2_PO_4_, 26 mM NaHCO_3_, 20 mM glucose, 1 mM pyruvate, and 1 mMTrolox) and kept at 35°C. For acute pharmacological treatments the ACSF contained one or more of the following substances: 0.1 μM tetrodotoxin [TTX; Sigma-Aldrich, St. Louis (MO), USA], 50 μM 4-aminopyridine (4-AP, Sigma-Aldrich), 20 μM bicuculline methiodide (Sigma-Aldrich), 10 μM SR-95531 [Gabazine; Tocris Bioscience, Ellisville (MO), USA], and 10 μM muscimol (Tocris Bioscience). For each treatment, data were obtained from treated slice cultures and untreated sister cultures (control).

For long-term activity blockade, TTX was added to the slice medium and renewed every day by exchanging 2/3 (500 μl) of the culturing medium (in controls and treated cultures). Treatment was continued until (and throughout) the imaging session, either 48 h or 7 days after the start of the treatment. The 7-day treatments started at DIV10. We found that 48 h of 4-AP treatment significantly affected the health of our organotypic cultures and we therefore did not include data from these treatments.

### Time-lapse two-photon microscopy

Time-lapse two-photon microscopy was used to monitor structural changes in GFP-labeled inhibitory boutons. We used a custom-built two-photon microscope, based on an Olympus IX70 microscope with a 40x, 1.2 NA, water immersion objective (Olympus, Tokyo, Japan). GFP was excited using a laser beam at 910 nm (Ti:Sapphire laser, Mai Tai HP, Spectra Physics, Darmstadt, Germany); for simultaneous excitation of GFP and Alexa 568 a wavelength of 950 nm was used. The emitted green and red fluorescence was detected using external photomultiplier tubes (R6357, Hamamatsu, Hamamatsu, Japan). 3D image stacks (spanning 100 × 100 μm in xy, and 40–50 μm in z; using 1024 × 1024 pixels in xy, and 0.5 μm steps in z; pixel dwell time 6 μs) were acquired every 30 min for a total experiment duration of 4 h.

Analysis of the bouton dynamics in individual axons was carried out in a semi-automated manner using software written in Matlab (Mathworks, Natick (MA), USA) and ImageJ (US National Institutes of Health). 3D coordinates of points along individual axons were selected at every time point using the CellCounter plugin (Kurt De Vos, University of Sheffield, Academic Neurology). These coordinates were used to generate a 3D intensity profile of the traced axons at every time point. For this purpose, the total intensity of a small area in the orthogonal plane was determined for each point of the axon (16 pixels on orthogonal line × 4 z-layers). For each image stack, 3–5 axons of >40 μm were usually included in the analysis.

The bouton threshold was determined for each time point in a two-step process. First, a local axon threshold was calculated for each image to distinguish the axon from the background (2 standard deviations (std) above mean intensity). Second, a local threshold (0.5 std above mean axon intensity) was calculated for all peaks of the 3D intensity profile, and the 33-percentile of the distribution of all local thresholds was taken as the bouton threshold for the entire axon. This bouton threshold was then used to identify boutons along the selected axon. We found this procedure to be optimal for detecting weak boutons, independent of bright neighboring boutons. Each image stack was visually examined to remove false positives and negatives. Only structures with 10 or more pixels above the bouton threshold were included. Bouton volumes were calculated as the summed intensity of all pixels above bouton threshold, divided by the bouton threshold. We used this normalization by bouton threshold because axons with different labeling intensity were often observed within the same two-photon image. It was verified that average bouton thresholds were not different between different treatments, indicating that overall GAD65-GFP expression levels were not affected by the acute activity manipulations. We verified that an alternative volume measurement (absolute number of pixels above bouton threshold) led to the same results. The coefficient of variation over all time points in bouton volume was calculated for each bouton individually by dividing the standard deviation of the bouton volume by its mean.

Instantaneous bouton loss (or gain) was determined by the number of lost (or gained) boutons divided by the total number of boutons present at each time point. Bouton density was determined as the average number of boutons per time point divided by the 3D axon length.

A growth coefficient was calculated for each bouton to quantify its growth or shrinkage during the imaging period: growth coefficient = (V2 − V1)/(V2 + V1), in which V1 and V2 are the average volumes of the first two (V1) and last two (V2) imaging time points. A positive value of the growth coefficient indicates bouton growth while a negative value indicates bouton shrinkage.

### Electrophysiology

Somatic whole-cell recordings were obtained from visually identified pyramidal cells in the CA1 area of the hippocampal slice cultures. Glass pipette electrodes (resistance: 2–4 MΩ) were pulled with a P-97 Flaming/Brown Micropipette puller (Sutter Instruments, Novato (CA), USA) from borosilicate capillaries (GC 150 F-10; Harvard Instruments, Holliston (MA), USA). The pipette internal solution contained 20 mMKCl, 100 mM cesium methylsulfonate, 10 mM HEPES, 4 mM Mg-ATP, 0.3 mM Na-GTP, 10 mM sodium phosphocreatine and 3 mM QX-314. Recordings were performed at 35°C in ACSF supplemented with pharmacological substances as described above.

To measure spontaneous synaptic currents, the reversal potentials for inhibitory and excitatory inputs were determined by clamping the cell to different potentials in 5 mV increments (measured values: −44.1 ± 1.3 mV for inhibitory currents, and 4.2 ± 1.7 mV for excitatory currents, *n* = 17 and 15 cells, respectively). For recording spontaneous excitatory currents, cells were clamped to the reversal potential of inhibitory currents, and vice versa. When bicuculline was present in the bath, excitatory currents were measured at −40 mV. Synaptic currents were recorded for 5 min, in 1.5-s sweeps. Data were acquired using custom-written LabView software (National Instruments, Austin (TX), USA).

Neurons were only included in the analysis when membrane potential was < −50 mV, input resistance >150 MΩ, and series resistance <25 MΩ. Data analysis was performed with custom-written Matlab software. Total excitatory and inhibitory charge was calculated as the integral of negative and positive currents in the respective recordings and given as total charge per second recording time.

### Immunohistochemistry

*Post-hoc* immunohistochemistry for pre- and postsynaptic markers and confocal imaging of inhibitory boutons were performed as described previously (Wierenga et al., 2008). For the c-fos immunohistochemistry, slice cultures were fixed in 4% w/v paraformaldehyde (30 min at 35°C, then 4 h at 4°C), washed extensively in 0.1 M phosphate buffer and removed from their cover slips to be processed as free-floating sections. Permeabilization and blocking were achieved by incubating the sections for 24 h at 4°C in 0.1 M phosphate buffer, 1% v/v Triton X-100 and 10% v/v goat serum. Primary antibodies (rabbit anti-c-fos 1:5000 (Merck, Darmstadt, Germany); mouse anti-NeuN 1:100 (Millipore, Billerica (MA), USA); chicken anti-GFP 1:1000 (Chemicon/Millipore)) were applied over night at 4°C in 0.1 M phosphate buffer, 1% v/v Triton X-100 and 5% v/v goat serum. After extensive washing with 0.1 M phosphate buffer, secondary antibodies (anti-chicken-Alexa 488, anti-rabbit-Cy3, anti-mouse-Alexa 633 [all 1:200, Molecular Probes, Invitrogen, Carlsbad (CA), USA)] were applied over night at 4°C in 0.1 M phosphate buffer and 5% v/v goat serum.

Confocal imaging was performed on a Leica LCS SP2 microscope. A z-projection over 30 μm of a 149 × 149 μm area in the CA1 region was analyzed for c-fos positive cells using the ITCN plugin for ImageJ (Thomas Kuo, JiyunByun, UCSB (CA), USA). The number of positive cells was corrected for differences in the total intensity of the NeuN staining in the same region to account for possible differences in neuron density.

### Local superfusion

To manipulate a small area of the slice culture, we adapted the technique described before (Veselovsky et al., 1996). Briefly, two pipettes, between which a super fusion solution was flowing through gravity flow, were lowered to the top of the cultured slice. The area affected by the super fusion was approximately 300 × 300 μm in size. The super fusion solution consisted of carbogenated ACSF with 10 μM Alexa 568 (Invitrogen) and, when indicated, 0.1 μM tetrodotoxin (TTX; Sigma-Aldrich). Time-lapse two-photon imaging was performed both inside and outside of the super fused area and analyzed as described above.

### Statistics

Data are reported as mean ± standard error unless stated otherwise. Statistical significance of differences between mean values for treated slices and matched untreated controls was tested using the two-tailed *t*-test. Multiple comparisons were made by an ANOVA followed by a *post-hoc* Tukey or Fisher test. Differences were considered significant if *p* < 0.05.

## Results

**Figure 1:**
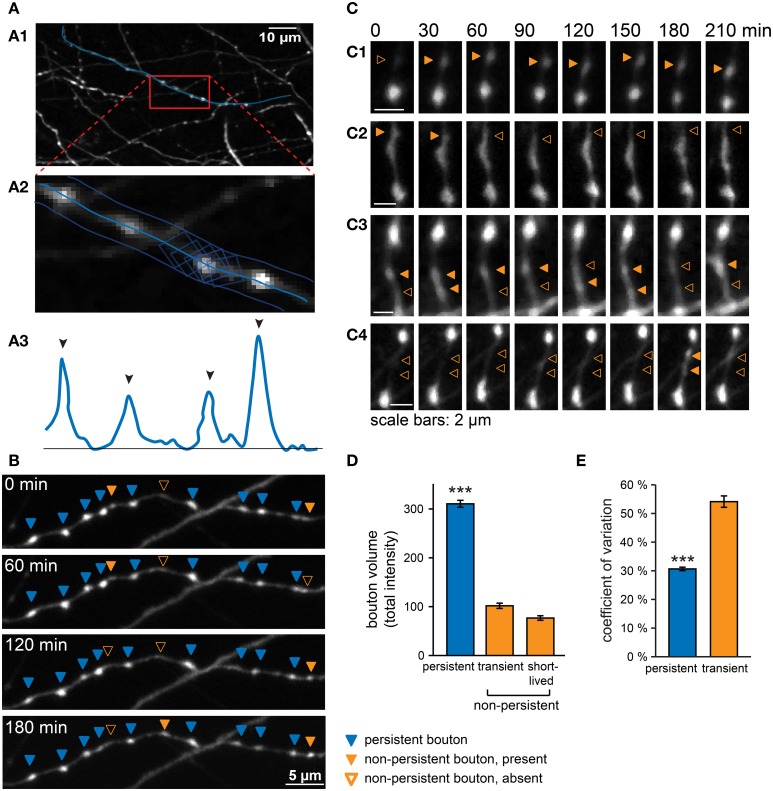
**Analysis of GABAergic bouton dynamics showing persistent and non-persistent GABAergic boutons. (A)** Boutons were located and analyzed in a semi-automatic manner with custom-written Matlab software. **(A1)** An axon was selected in the raw image data, and a3D-curve was fitted through the axon. **(A2)** The summed intensity in orthogonal planes along the fitted curve was determined and used to generate a 3D intensity profile of the axon **(A3)**. Black arrowheads indicate automatically detected peaks of the intensity profile above threshold. **(B)** Time-lapse imaging data from a GABAergic axon, showing persistent (blue arrowheads), and non-persistent boutons (closed orange arrowheads: bouton is present; open orange arrowheads: bouton is absent). **(C)** Examples of non-persistent boutons. **(C1)** A new bouton is gained at the second time point and persists until the end of the imaging period. **(C2)** A bouton disappears at the third time point. **(C3)** Two boutons disappearing and re-appearing. **(C4)** Two short-lived boutons. **(D)** Average bouton volume (determined by total intensity, see Materials and Methods) of persistent, transient, and short-lived boutons under control conditions. Significance level: ^***^p < 0.005 (ANOVA; *post-hoc* Tukey). **(E)** Coefficient of variation (CV) of bouton volume for persistent and transient boutons under control conditions (total time = 4 h; 8 time points). Images were filtered for illustration purposes. Analysis was always performed on raw images.

We performed time-lapse microscopy in organotypic hippocampal cultures from GAD65-GFP mice to characterize the structural dynamics of presynaptic GABAergic boutons. In these mice, GFP is expressed in a known subset of dendritically-targeting interneurons comprising about 20% of the entire interneuron population (Wierenga et al., 2010). High-resolution two-photon image stacks were acquired at 30 min intervals in the *stratum radiatum* of the CA1 region over a total period of 4 h. Individual axons were identified, and boutons along the axons were located at every time point (Figure 1A). Potential boutons were automatically detected as peaks in the 3D intensity profile and then verified by visual inspection in the original image stacks. Only boutons above threshold were included in the analysis (see Materials and Methods). For simplicity, we use the term “bouton” for any thickening of the axonal shaft that satisfied our detection criteria thereby including presynaptic terminals as well as non-synaptic varicosities (see below).

The majority of boutons was present at every time point throughout the imaging period (~80% of boutons present at a single time point), i.e. for at least 4 h (“persistent boutons”). The remaining boutons were present in only a subset of time points during the imaging period. On average, these non-persistent boutons were present for ~40% of the imaging period, but the time course of appearance, disappearance and reappearance varied widely between individual boutons (Figure 1B). Only in a few cases we unambiguously observed a new bouton appearing (Figure 1C1), or an existing bouton disappearing (Figure 1C2). Merging and splitting of boutons (Dobie and Craig, 2011)occurred occasionally (1.5 ± 0.2 merging and 1.8 ± 0.2 splitting events per 100 μm axon in 4 h). We did not observe any obvious translocation of persistent boutons in our organotypic cultures (Dobie and Craig, 2011). In the majority of cases boutons were detected at variable time points at the same axonal position with often large fluctuations in bouton size (Figure 1C3). In addition, we observed some boutons that occurred only once or twice within the entire imaging period (Figure 1C4). Boutons that showed the latter behavior formed ~50% of the total population of non-persistent boutons, but because of their sporadic occurrence, they made up only ~4% of all boutons along an axon at a single time point. Within a limited imaging period it is impossible to unequivocally determine bouton category based on their presence or absence, but for reasons clarified below, we termed the latter group “short-lived” boutons and treat them separately from the other non-persistent boutons (termed “transient” boutons), which occurred multiple times during the imaging period.

We next determined the bouton volumes of the different types of boutons. Persistent boutons had on average ~3-fold larger volumes compared to transient and short-lived boutons (Figure 1D). We noticed that the volumes of all boutons, including persistent boutons, showed remarkable variation over time (Figure 1E; see also individual examples in Figures 1B,C). Volume variations were not correlated between neighboring boutons and therefore likely not due to experimental variations in our measurements, but reflecting true biological variation. This suggests that the presynaptic content of inhibitory synapses strongly fluctuates over a period of tens of minutes to hours.

### Persistent and non-persistent GABAergic boutons differ with respect to their synaptic markers

**Figure 2:**
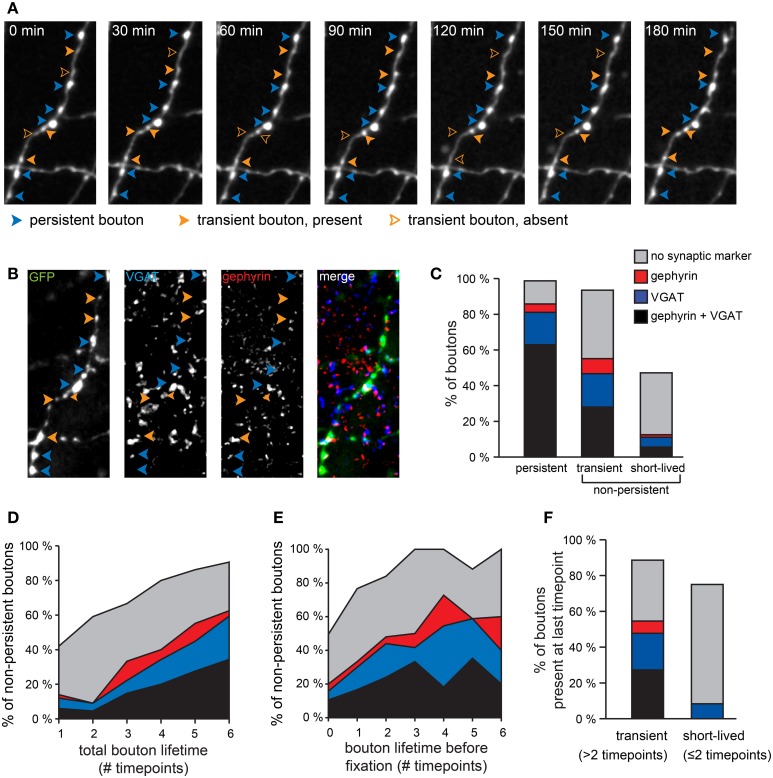
**Different synaptic marker content for persistent and non-persistent GABAergic boutons. (A)** Persistent and non-persistent boutons along an individual GABAergic axon were identified and analyzed. Blue arrowheads: persistent boutons; filled and open orange arrowheads: present and absent non-persistent boutons, respectively. **(B)** Immunohistochemistry [vesicular GABA transporter (VGAT, blue), gephyrin (red), GFP (green)] of the same axon. **(C)** Percentage of boutons with synaptic markers (color code as in **B**) for each bouton category. Data from 470 persistent, 123 transient, and 72 short-lived boutons from two separate experiments. **(D)** Fraction of non-persistent boutons co-localizing with synaptic markers (color coded) as a function of bouton lifetime, determined as the total number of time points that the bouton was present during the imaging period. **(E)** Same as **(D)**, but now as a function of lifetime before fixation. **(F)** Synaptic labeling of transient (*n* = 88) and short-lived (*n* = 12) boutons that were present at the last time point before fixation. Percentages do not add to 100%, because in some cases boutons that were identified during the two-photon imaging period were not present in the *post-hoc* immunostaining images, usually because they had disappeared before the end of the imaging period.

In our previous work, we have shown that the vast majority of persistent boutons are associated with markers for GABAergic synapses and are likely to represent established inhibitory synapses (Wierenga et al., 2008). We hypothesized that non-persistent boutons could represent synapses in the process of assembly or during disassembly (Wierenga et al., 2008), but they could also reflect “stop-sites” of transport vesicles containing synaptic material (Sabo et al., 2006) or aggregation spots for other material transported along the axon (Goldstein et al., 2008; Cai and Sheng, 2009). To address the molecular features of non-persistent boutons, we performed *post-hoc* immunohistochemistry of hippocampal slices following the time-lapse two-photon imaging sessions and correlated synaptic markers of individual boutons with their previous dynamic behavior during two-photon time-lapse imaging (Figures 2A,B). We used the presynaptic vesicular GABA transporter (VGAT) and the postsynaptic scaffolding molecule gephyrin as markers of GABAergic synapses. In addition, we used antibodies against GFP to identify previously imaged GAD65-GFP positive axons (Wierenga et al., 2008). As expected, the vast majority of persistent boutons (86%) were associated with at least one synaptic marker and 63% of them showed both VGAT and gephyrin labeling. In contrast, a much smaller fraction of the non-persistent boutons was associated with one or both of these synaptic markers (Figure 2C). Interestingly, synaptic labeling of non-persistent boutons seemed to be determined by the total lifetime of a bouton (i.e. the total number of (consecutive or non-consecutive) time points the bouton was observed during the imaging period; Figure 2D), rather than by its lifetime before fixation (i.e. number of consecutive time points before fixation; Figure 2E). This suggests that the acquisition of synaptic markers at a specific location along the axon is determined by the prevalence of local bouton occurrence. The actual presence of a bouton at the time of fixation appears to be of secondary importance.

Most of the non-persistent boutons that were present for only one or two time points during the imaging period were not associated with synaptic markers, even when they were observed right before fixation (Figure 2F). We have termed these boutons “short-lived” and we suggest that the majority of them do not represent synaptic boutons. They probably represent snapshots of movement along the axon (Sabo et al., 2006; Bury and Sabo, 2011; Dobie and Craig, 2011) and their precise location may be merely determined by the time when the image was taken. In contrast, a substantial fraction of non-persistent boutons that were observed for at least three time points (not necessarily consecutive), were associated with synaptic markers. We termed these “transient boutons” (Figure 2C). We have previously observed transient inhibitory boutons at axon-dendrite crossings, which were often associated with the formation of a new inhibitory contact at the same location on a longer timescale (Wierenga et al., 2008). This suggests that at least a subset of transient boutons that were observed for at least 3 time points during our imaging period are of synaptic nature and they could be involved in formation and disassembly of inhibitory synapses.

### Synaptic markers of persistent boutons

**Figure 3:**
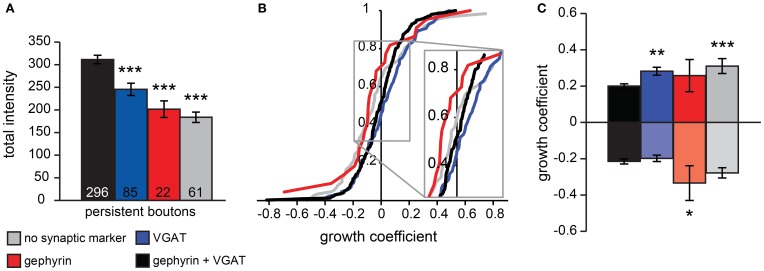
**Analysis of persistent bouton volumes. (A)** Average volume of persistent boutons separated by synaptic markers (determined from *post-hoc* immunohistochemistry). Numbers at the bottom of bar graphs indicate the number of boutons in each group. **(B)** Cumulative distribution of growth coefficient values for persistent boutons separated by synaptic labeling. Inset shows the crossing of the curves with the vertical axis, indicating the fraction of shrinking boutons for each group. **(C)** Average growth coefficient of the 25% most growing and shrinking boutons in each group. Significance levels: ^*^p < 0.05, ^**^p < 0.01, ^***^p < 0.005 (ANOVA, *post-hoc* Fisher; only differences with the black bars are shown).

Even though the majority of persistent boutons presumably reflect inhibitory synapses, a subset of persistent boutons was associated with no or only one synaptic marker. We wondered whether those boutons could represent immature synapses that were recently formed and/or incomplete synapses about to be disassembled. To address this question we measured average bouton volumes of all persistent boutons and correlated bouton volumes with synaptic markers. We found that boutons that were associated with both synaptic markers were generally larger than boutons without detectable VGAT or gephyrin (Figure 3A). This suggests that differences in synaptic markers reflect separate bouton populations.

To examine if synaptic marker labeling of boutons could be correlated to processes of bouton formation or disassembly, we examined the growing or shrinking behavior of persistent boutons during the time-lapse imaging period by determining a growth coefficient (between −1 and 1) for each bouton. The growth coefficient was positive for growing and negative for shrinking boutons (see Materials and Methods). On average, the population of persistent boutons did not substantially grow or shrink during the imaging period (mean growth coefficient was −0.002 ± 0.009), but individual persistent boutons did show significant growth or shrinkage. We determined the growth coefficients for each bouton and constructed cumulative plots for the population of persistent boutons with different synaptic labels (Figure 3B). The crossing of the vertical axis indicates the fraction of shrinking boutons within the population. We found that there were relatively many shrinking boutons in the population of boutons with only gephyrin and a large fraction of growing boutons in the population of boutons with only VGAT. Next, we determined the growth coefficients of the 25% most growing and shrinking boutons for each type of bouton (Figure 3C). This analysis showed that boutons associated with both pre- and postsynaptic markers had the largest volumes (Figure 3A) and the smallest volume changes (Figure 3C), in agreement with the interpretation that they represent established, stable GABAergic synapses. Boutons with only postsynaptic gephyrin showed significantly stronger shrinkage, while boutons with only VGAT showed stronger growth compared to boutons with both synaptic markers. The small fraction of persistent boutons (13%), for which we could only detect the volume label (GFP) in *post-hoc* confocal microscopy (grey in Figures 2, 3), showed relatively strong growth and shrinkage (Figure 3C). We speculate that this population of small boutons reflects assembling and degrading boutons with low levels of synaptic markers. These data strongly suggest a correlation between the synaptic markers of inhibitory boutons and their growing or shrinking behavior.

### Network activity affects GABAergic bouton dynamics

**Figure 4:**
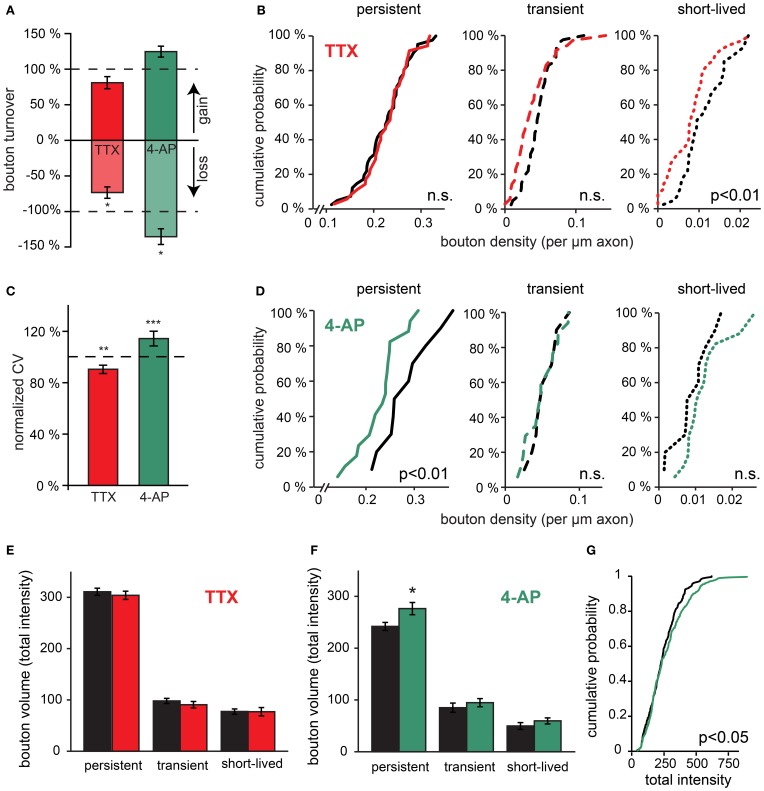
**Rapid effects of changes in network activity on bouton turnover. (A)** Normalized bouton turnover of inhibitory axons during TTX and 4-AP treatment (averages per time point). Bouton turnover was defined as the fraction of boutons gained or lost between two consecutive imaging time points. Control values: bouton gain = 8.0 ± 0.2%, bouton loss = 8.7 ± 0.4%. **(B)** Cumulative distribution of the density of persistent, transient and short-lived boutons following TTX treatment. Data from 41 control and 34 TTX-treated axons (Student's *t*-test). **(C)** Normalized coefficient of variation (CV) of bouton volume for persistent boutons during 4 h of TTX and 4-AP treatment. **(D)** Cumulative distribution of the density of persistent, transient and short-lived boutons following 4-AP treatment. Data from 10 control and 17 4-AP treated axons (Student's *t*-test). **(E)** Average bouton volume of persistent, transient and short-lived boutons during 4 h control (black bars) or TTX treatment (grey bars). **(F)** Same for 4 h 4-AP treatment (grey bars). **(G)** Cumulative distributions of persistent bouton volumes (measured as total intensity) in control (black) and 4-AP treatment (grey). Significance levels: ^*^p < 0.05; ^**^p < 0.01; ^***^p < 0.001 (Student's t-test between treatment and its control).

The described dynamics of inhibitory boutons suggest axonal transport and exchange of bouton content between immature as well as established inhibitory synapses. Exchange of presynaptic material along the axonal shaft of excitatory axons has been proposed to play an important role during synaptic plasticity (Krueger et al., 2003; McAllister, 2007; Staras et al., 2010). To assess the possible role of axonal dynamics in activity-regulated inhibitory plasticity, we asked whether the dynamics of inhibitory boutons are affected by changes in network activity. We assessed instantaneous bouton turnover by determining the fraction of boutons that were gained or lost between each two consecutive time points (30 min) using time-lapse two-photon imaging. In control slices, 8.0 ± 0.2% of boutons appeared new between consecutive time points and 8.7 ± 0.4% of boutons were lost (represented as the fraction of boutons present at a single time point). In slices in which network activity was reduced with 0.1 μM TTX bouton turnover was significantly lower (Figure 4A; bouton gain: 80.9 ± 8.5%, bouton loss: 73.4 ± 8.1% of control values), while enhancing network activity with 50 μM 4-AP increased bouton turnover relative to control values (Figure 4A; bouton gain: 124.5 ± 7.7%, bouton loss: 135.4 ± 11.0% of control values). For both treatments, only the effect on bouton loss was significant.

Changes in bouton dynamics were examined in more detail by determining the density of persistent, transient, and short-lived boutons per axon in control and treated slices. We found that TTX treatment did not affect the density of persistent boutons (102 ± 5% of control; *p* = 0.7), but the density of transient and short-lived boutons was reduced (Figure 4B; 78 ± 9% of control; *p* < 0.05; for transient and short-lived boutons together). In view of our interpretation above, this implies that activity blockade did not affect established inhibitory synapses (represented by persistent boutons), but the reduction of non-persistent boutons suggested a rapid down regulation of axonal transport and exchange between boutons when activity was decreased. In contrast, treatment with 4-AP induced a significant reduction in the number of persistent boutons (80 ± 6% of control; *p* < 0.01). The density of short-lived boutons appeared to increase, but this did not reach significance (Figure 4D). This suggests destabilization of GABAergic synapses during 4-AP treatment and possibly enhanced transport within inhibitory axons. Taken together, our data show that regulation of GABAergic bouton dynamics in response to changes in network activity can occur within just a few hours, and that increasing or decreasing activity affects persistent and non-persistent boutons differentially.

### Activity-dependent effects on bouton volumes

It has been suggested that the size of presynaptic boutons is correlated with the strength of the synapses (Schikorski and Stevens, 1997; Holderith et al., 2012). We were wondering if bouton volumes were affected by activity treatments, possibly reflecting changes in synaptic strength. We found that average bouton volumes were generally not affected by the 4-h activity manipulations (Figures 4E,F), except for a small but significant increase in volume of large persistent boutons in response to acute 4-AP treatment (Figure 4G). The latter observation possibly reflects a specific strengthening of large boutons in 4-AP (Peng et al., 2010). Despite the minimal change in average bouton volumes, we found that the volume variations of GABAergic boutons were markedly modified by changes in network activity (Figure 4C). When activity was reduced by TTX, the coefficient of variation of persistent bouton volumes was significantly decreased (90 ± 3% of control), whereas it was increased under conditions of enhanced activity in 4-AP (114 ± 6% of control). These activity-dependent changes in bouton volume fluctuations over time were in the same direction as the observed changes in bouton dynamics (Figure 4A). This suggests that both parameters are governed by the exchange of bouton content between inhibitory synapses along the axonal shaft and that this exchange is rapidly affected by ongoing network activity.

### Prolonged manipulations of network activity

**Figure 5:**
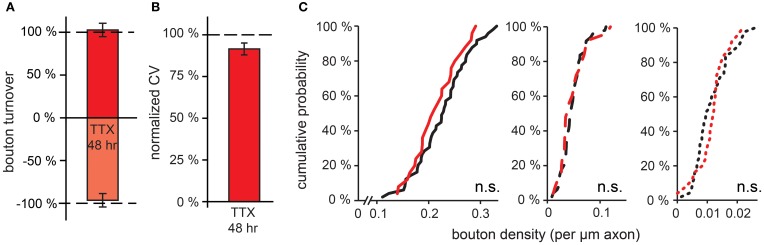
**Effect of prolonged (48 h) reduction in network activity on GABAergic bouton dynamics. (A)** Normalized bouton turnover after 48 h TTX treatment. Control values: bouton gain = 8.1 ± 0.2%, bouton loss = 8.7 ± 0.3%. **(B)** Normalized coefficient of variation (CV) of bouton volume for persistent boutons after 48 h TTX treatment. **(C)** Cumulative distribution of the density of persistent, transient and short-lived boutons following 48 h TTX treatment. Data from 49 control and 25 TTX-treated axons (Student's *t* test).

We were wondering whether the activity-induced effects on GABAergic bouton turnover would eventually translate into changes in GABAergic bouton density after more prolonged changes in network activity. We subjected hippocampal organotypic cultures to 48 h treatment with TTX to decrease network activity before performing our time-lapse imaging experiments. We found that after this treatment bouton turnover was similar to control values (Figure 5A; bouton gain: 102.7 ± 7.9%, boutons loss: 96.5 ± 7.9% of control values). Bouton volumes were not different between treated slices and control and also the CV of persistent bouton volumes was not significantly different from control values (Figure 5B; 91.5 ± 3.5% of control values). Furthermore, no difference in the density of transient boutons or short-lived events was observed (Figure 5C). Even when we prolonged the TTX treatment to 7 days, we did not detect any difference in bouton density of inhibitory axons (control: 29 ± 1, TTX: 27 ± 1 boutons per 100 μm axon). This indicates that in hippocampal organotypic cultures, prolonged reduction of the activity level does not result in changes in the number of inhibitory synapses.

### Activity-induced structural GABAergic plasticity requires activation of GABA_A_ receptors

**Figure 6:**
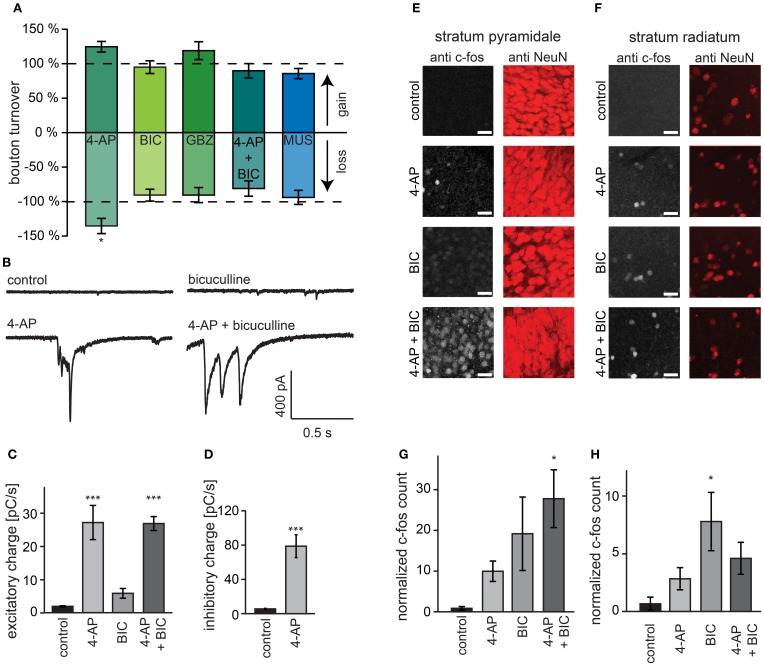
**Activation of GABA_A_ receptors is required for bouton turnover enhancement. (A)** Normalized GABAergic bouton turnover following acute treatment with 4-AP (50 μM), bicuculline (BIC; 20 μM), gabazine (GBZ; 10 μM), co-application of bicuculline and 4-AP (4-AP + BIC), and muscimol (MUS; 10 μM). Number of axons: 4-AP: 17 treated and 10 control axons; bicuculline: 22 treated and 41 control axons; gabazine: 16 treated and 13 control axons; AP + bicuculline: 14 treated and 14 control axons; muscimol: 15 treated and 15 control axons. Significance levels: ^*^p < 0.05 (Students *t*-test between treatment and its control). **(B)** Whole-cell recordings of spontaneous excitatory currents from visually identified CA1 pyramidal cells during treatment with indicated agent. **(C)** Average total charge of spontaneous excitatory postsynaptic currents in CA1 pyramidal cells. **(D)** Average total charge of spontaneous inhibitory postsynaptic currents in CA1 pyramidal cells during treatment. **(E,F)** Immunohistochemistry for the immediate early gene c-fos in CA1 stratum pyramidale **(E)** and *stratum radiatum* **(F)** in hippocampal organotypic cultures after 4 h treatment. **(G,H)** Number of c-fos positive cells in the CA1 stratum pyramidale **(G)** and *stratum radiatum* **(H)** corrected for the total intensity of the NeuN staining. Significance levels: ^*^p < 0.05, ^***^p < 0.005 (ANOVA, *post-hoc* Tukey test).

**Figure 7:**
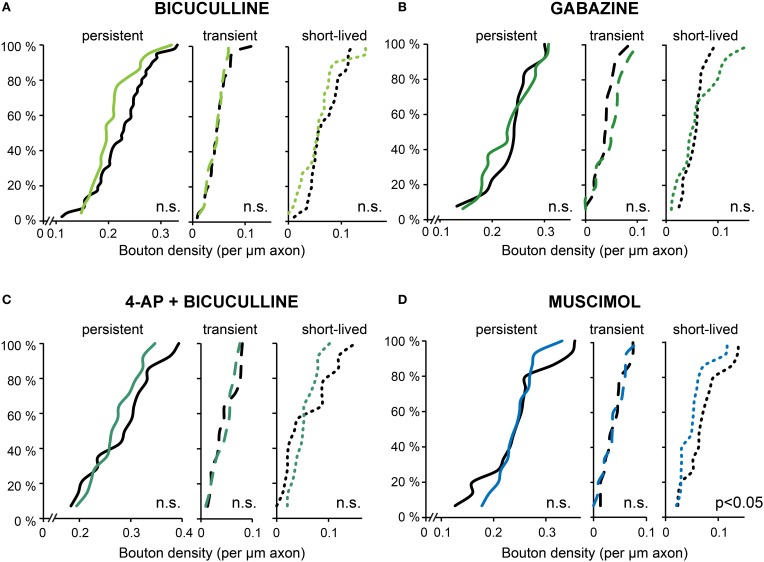
**Bouton densities after different activity manipulations. (A–D)** Cumulative distribution of the density of persistent, transient and short-lived boutons following bicuculline **(A)**, gabazine **(B)**, 4-AP + bicuculline **(C),** and muscimol **(D)** treatment. Number of axons: bicuculline: 22 treated and 41 control axons; gabazine: 16 treated and 13control axons; AP + bicuculline: 14 treated and 14 control axons; muscimol: 15 treated and 15control axons (Student's *t*-test).

As an alternative to 4-AP we used bicuculline for increasing activity in our organotypic cultures. To our surprise and in strong contrast with our earlier findings for 4-AP, we found that acute treatment with bicuculline did not affect GABAergic bouton turnover (gain: 94.9 ± 9.2%, loss: 90.7 ± 8.6% of control values; Figure 6A) or density (Figure 7A). We verified with the highly specific GABA_A_ receptor antagonist gabazine (10 μM SR-95531) that the lack of effect was not due to an off-target effect of the GABA_A_ receptor blocker bicuculline (Figures 6A, 7B; bouton gain: 118.8 ± 13.0%, bouton loss: 90.6 ± 11.0% of control value). A possible explanation for the difference in the response to 4-AP and bicuculline would be that the network activity level induced by GABA_A_ receptor antagonists was lower than that induced by 4-AP. We therefore co-applied 4-AP and bicuculline in an attempt to enhance network activity maximally. However, the combined application did also not change GABAergic bouton turnover (gain: 89.6 ± 10.4%, loss: 81.1 ± 11.1% of control values; Figure 6A)or density (Figure 7C).

However, it has been reported that under certain circumstances, 4-AP can reverse the driving force for chloride (Lamsa and Taira, 2003), in which case bicuculline would reduce network activity relative to treatment with 4-AP alone. We therefore measured the level of network activity in our slices during the pharmacological treatments using two independent methods. First, we recorded spontaneous excitatory and inhibitory currents in hippocampal CA1 pyramidal cells and determined total synaptic input as a measure of the activity of the slice (Maffei et al., 2004; Dani et al., 2005). We found that although bicuculline and 4-AP both enhanced excitatory synaptic input to the neurons, the effect was much stronger in 4-AP. Importantly, when slices were treated with the combination of 4-AP and bicuculline, the increase in synaptic current was similar to the increase observed with 4-AP alone (Figures 6B,C). In addition, 4-AP treatment strongly enhanced inhibitory currents onto CA1 pyramidal neurons (~14-fold increase; Figure 6D). Specifically large, synchronous events were found in both 4-AP and in 4-AP+bicuculline treated slices, which were never observed in control and bicuculline-treated slices (data not shown). Second, using immunohistochemistry, we measured c-fos activation after 4 h treatment with 4-AP, bicuculline and their combination. C-fos immunohistochemistry has been used frequently to label recently active neurons (Dragunow and Faull, 1989; Tischmeyer and Grimm, 1999; Schoenenberger et al., 2009). We found that 4-AP and bicuculline individually increased c-fos levels in the entire CA1 area compared to control (Figures 6E–H). Combining 4-AP and bicuculline treatment resulted in c-fos levels that were even higher than the sum of the individual treatments.

These observations suggest that the differential effect on bouton dynamics of treatment with 4-AP and GABA_A_ receptor antagonists cannot be explained by a differential effect on the activity level, and suggest a requirement of GABA_A_ receptor activation for the increase in bouton dynamics. To examine whether activation of GABA_A_ receptors can enhance bouton dynamics by itself, we applied the GABA_A_ receptor agonist muscimol, but this did not induce an increase in inhibitory bouton density or turnover (Figures 6A, 7D). Instead, muscimol induced a small decrease in the density of short-lived boutons (Figure 7D), possibly because of decreased neuronal activity in the slice (compare with Figure 4B). These findings show that the activation of GABA_A_ receptors is necessary, but not sufficient, to induce the rapid increase in bouton dynamics.

### Blocking network activity in a restricted area also reduces inhibitory bouton dynamics

**Figure 8:**
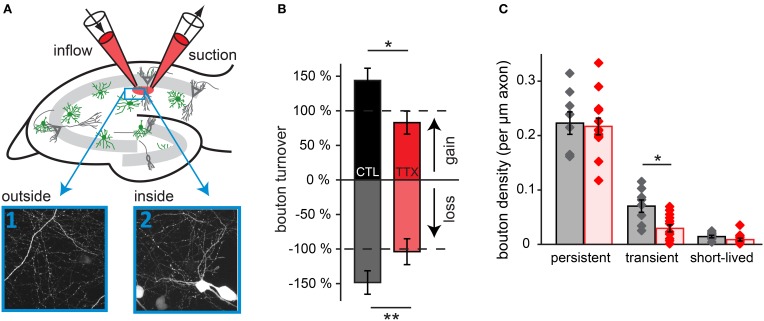
**Blocking activity in a small region of the slice decreases bouton turnover. (A)** Experimental setup for local super fusion experiments: a small region in the CA1 area of the hippocampal slice culture is super fused with ACSF (labeled with Alexa 568) containing 0.1 μM TTX or control ACSF. High-resolution two-photon images were taken inside and outside of the super fusion spot. **(B)** Normalized bouton turnover during perfusion with TTX and control solution. **(C)** Bouton density for each bouton category during perfusion with TTX and control solution. Control super fusion: 8 axons inside, 8 outside; TTX super fusion: 13 axons inside, 12 outside. Significance levels: ^*^p < 0.05, ^**^p < 0.01 (ANOVA, *post-hoc* Tukey test).

Finally, we addressed the question whether changes in inhibitory bouton dynamics require network-wide changes in activity (Hartman et al., 2006; Goold and Nicoll, 2010) or whether more local changes suffice (Liu, 2004; Di Cristo et al., 2007; Peng et al., 2010). For this purpose, we adapted a local super fusion technique (Veselovsky et al., 1996) using two pipettes that were lowered on top of the organotypic slice culture. In this way, neuronal activity in a part of the CA1 area (~300 × 300 μm) was reduced by super fusion with a solution containing 0.1 μM TTX and 10 μM Alexa 568 in ACSF. The Alexa dye highlighted the manipulated region for optical control during the experiment (Figure 8A). Images were taken both inside and outside the manipulated region, and experiments and data analysis were performed as described above. Super fusion with control solution already increased bouton turnover by 40–50% compared to control (no super fusion: gain: 7.4 ± 0.9% (*p* = 0.06), loss: 6.5 ± 0.8% (*p* < 0.05); super fusion with control solution: gain: 10.6 ± 1.3%, loss: 9.7 ± 1.1%). However, despite this general increase, super fusion with a TTX-containing solution significantly decreased bouton turnover inside the super fusion spot (gain: 6.1 ± 1.2%, loss: 6.8 ± 1.2%) compared to super fusion with the control solution (Figure 8B; gain: 10.6 ± 1.3%, loss: 9.7 ± 1.1%). In addition, the density of transient boutons was decreased to less than 50% in regions super fused with 0.1 μM TTX, if compared to the region super fused with the control solution (Figure 8C; control: 8.5 ± 1.2 transient boutons per 100 μm axon, TTX: 3.8 ± 0.8 transient boutons per 100 μm axon; *p* < 0.01).

Our data indicates that super fusion with TTX decreased bouton turnover to at least a similar extent as bath application of TTX. Reducing activity in a restricted area of the CA1 network can elicit the same structural GABAergic plasticity as manipulation of the entire slice, suggesting that activity-dependent bouton dynamics could be locally regulated. However, these experiments do not exclude the involvement of factors secreted from neighboring cells that are not directly contacted by the GABAergic axon. To address these issues in the future, manipulating the activity of individual cells will be necessary.

## Discussion

We have used time-lapse two-photon imaging to characterize structural dynamics of GABAergic boutons in the dendritic layers of the CA1 region in organotypic hippocampal cultures from GAD65-GFP mice. Under baseline conditions, ~20% of inhibitory boutons are transient in nature, and include processes in which inhibitory synapses are being formed and disassembled. Our data show that inhibitory boutons, even when part of stable inhibitory synapses, are highly dynamic structures displaying large volume variations over time. Inhibitory axons are continuously probing potential locations for forming new synapses by assembling and disassembling boutons along the shaft. Furthermore, our data show that neuronal activity in the surrounding tissue affects the dynamics of these structural changes. Prolonged changes in neuronal activity level are known to induce changes in synaptic strength or number. Our data suggest that for inhibitory connections such changes are preceded, and possibly partially manifested, by transient changes in bouton dynamics, modifying the exploration of potential synaptic locations.

### Bouton dynamics

We report a wide range of inhibitory bouton dynamics: strong fluctuations in bouton volumes, transient and often repeated appearances of boutons at a specific axonal location as well as merging and splitting of boutons. Most notably, our analysis of bouton volumes showed that, despite their uninterrupted presence at a stable position during the imaging period, persistent boutons showed significant variations in bouton volume over a period of several hours, even under baseline conditions. This implies that inhibitory boutons, even when part of mature synapses, are not fixed and stable entities, but are shaped by ongoing dynamic exchange along the axon. For excitatory axons, it has been shown that presynaptic terminals are continuously exchanging presynaptic material and vesicles, by transport of small clusters as well as by passive diffusion through the axonal shaft (Chi et al., 2001; Darcy et al., 2006; Sabo et al., 2006; Trigo et al., 2006; Fernandez-Alfonso and Ryan, 2008; Staras et al., 2010; Bury and Sabo, 2011). Our data, together with recent data by others (Dobie and Craig, 2011; Fu et al., 2012; Kuriu et al., 2012; Wu et al., 2012), shows that exchange between presynaptic boutons is a general feature of inhibitory axons of different interneuron types and in different brain areas and preparations. The absolute level of bouton turnover and volume dynamics likely varies between dissociated and organotypic cultures and *in vivo*, and may depend on other experimental differences such as age. We observed that volume variations were much more pronounced in transient boutons compared to persistent boutons (Figure 1E), which could not simply be explained by their reduced volume. This suggests that exchange between immature synapses is strong, possibly reflecting weak anchoring of presynaptic content (Brigidi and Bamji, 2011; Sun and Bamji, 2011)or enhanced competition compared to more mature synapses (Krueger et al., 2003; Staras et al., 2010; Dobie and Craig, 2011; Fu et al., 2012). Bouton volume correlates with synaptic strength, when a population of boutons is considered but for individual synapses this relation is less clear (Minerbi et al., 2009; Dobie and Craig, 2011). We do not know whether the volume fluctuations observed in persistent boutons are translated in fluctuations in synaptic strength at individual inhibitory synapses.

### Transient boutons

The observed multiple recurrences of boutons at specific locations along the axonal shaft suggest that these locations are somehow available to accommodate synapses. Transient boutons showed small volumes and large volume variations and often were associated with only the pre- or only the postsynaptic marker. Previously, we have shown that new GABAergic synapses are formed by the emergence of new boutons along the axonal shaft and that transient appearances of inhibitory boutons at axon-dendrite crossings often resulted in the formation of new inhibitory contacts on a longer time scale (Wierenga et al., 2008). Here, we extend these findings by showing that locations where transient boutons appeared were often characterized by synaptic markers, strongly suggesting that such locations represent potential synaptic sites intermittently occupied by immature, or incomplete, synapses. Our analysis of synaptic markers of growing and shrinking boutons (Figure 3) support the notion that, similar to what has been reported for glutamatergic synapses (Friedman et al., 2000; Okabe et al., 2001), changes at the presynaptic side of GABAergic synapses precede postsynaptic changes during synapse assembly as well as during synapse disassembly (Wierenga et al., 2008; Dobie and Craig, 2011). Available axon-dendrite crossings could be marked by the presence of specific adhesion molecules (Varoqueaux et al., 2004; Chubykin et al., 2007; Huang and Scheiffele, 2008; Fu and Huang, 2010), although their role in synapse formation is currently not entirely clear. When synaptic material is halted or captured at these locations, they could become visible as transient boutons in two-photon microscopy. Here, neurotransmitter release could occur even in the absence of an active zone (Krueger et al., 2003; Coggan et al., 2005; Ratnayaka et al., 2011)and could induce further recruitment of molecular components of pre- or postsynaptic specializations.

### Activity-dependent changes in bouton dynamics

Our data indicate that the appearance and disappearance of boutons along inhibitory axons is rapidly affected by neuronal activity. Interestingly, we found that activity did not simply regulate the rate of exchange between boutons. The quantitative analysis allowed us to identify specific changes in subsets of boutons when neuronal activity was altered. Blocking activity with TTX specifically reduced non-persistent boutons, while leaving the persistent boutons unaffected. This suggests that blocking activity induces a reduction in the probing of new potential inhibitory synapse locations. Enhancing activity had the opposite effect: it resulted in a rapid decrease in the density of persistent boutons, suggesting that a subset of inhibitory boutons were destabilized. The concurrent increase in the density of short-lived boutons suggests increased axonal transport and exchange between neighboring inhibitory synapses, possibly reflecting increased competition for limited (presynaptic) resources (Govindarajan et al., 2011; Ratnayaka et al., 2011). We propose that the rapid adjustment of bouton dynamics and exchange of bouton content along the inhibitory axon during changes in network activity could serve to facilitate presynaptic changes (Hartman et al., 2006; Peng et al., 2010; Rannals and Kapur, 2011; Kuriu et al., 2012). When more presynaptic material would become available over time [e.g. by increased protein synthesis (Huang and Scheiffele, 2008)], an increased demand for presynaptic resources could lead to an overall strengthening of synapses. Indeed, the small, but significant increase in volume of large boutons that we observed in 4-AP (Figure 4G) would be consistent with such a growth-through-competition mechanism between boutons. Interestingly, it was recently reported that destabilization of persistent boutons together with increased volume variations also occurs in aging (Grillo et al., 2013).

We did not detect an effect of activity manipulation over a longer period on inhibitory bouton dynamics or density. These data are in agreement with earlier findings in cultures (Rutherford et al., 1997; Kilman et al., 2002; Hartman et al., 2006; Swanwick et al., 2006; Kuriu et al., 2012), where it was shown that long-term activity manipulations induces changes in the strength of inhibitory synapses without structural changes in GABAergic axons (but see Peng et al., 2010). In organotypic cultures, it was shown that activity blockade can prevent the developmental increase in bouton density (Marty et al., 2000, 2004; Chattopadhyaya et al., 2004), but to our best knowledge, an activity-dependent loss of established inhibitory boutons has not been reported. *In vivo*, several studies have found that a reduction in sensory input is accompanied by a decrease in inhibitory bouton density (Marik et al., 2010; Chen et al., 2011; Keck et al., 2011; van Versendaal et al., 2012). Although these differences may reflect experimental limitations of the culturing systems, they also underscorethe fundamental difference between global blockade of network activity with TTX in cultures, and a reduction of the sensory input to an otherwise normal network *in vivo*.

### The role of GABA_A_ receptors

Our data suggests that an increase in activity—and therefore GABA release—may be sensed by GABA_A_ receptors, resulting in enhanced axonal dynamics in inhibitory axons. Interestingly, the complementary effect, enhanced bouton stability in the absence of GABA, has recently also been reported (Wu et al., 2012). At this moment, we have no means to distinguish between pre- or postsynaptic GABA_A_ receptor signaling. Although presynaptic GABA_A_ receptors have been described in the central nervous system (Trigo et al., 2008), there is currently no experimental evidence for GABA_A_ receptors at inhibitory axons or synaptic terminals in the hippocampal CA1 area. So it seems likely that the changes we observe are due to postsynaptic effects of GABA_A_receptors. It has been shown that activation of postsynaptic GABA_A_ receptors plays an important role in the maturation of inhibitory synaptic contacts (Fritschy et al., 2006; Chattopadhyaya et al., 2007; Huang and Scheiffele, 2008). Here we found that activation of GABA_A_ receptors specifically plays a role in enhancing axonal dynamics (Figure 6A). The dynamics of postsynaptic gephyrin and GABA_A_ receptor clusters have been shown to be regulated by postsynaptic calcium influx (Hanus et al., 2006; Bannai et al., 2009). This then suggests that the pre- and postsynaptic sides of inhibitory synapses may be regulated independently using different activity sensors. The precise mechanism by which (postsynaptic) GABA_A_ receptors change presynaptic boutons dynamics is currently unclear. Potentially, activation of GABA_A_ receptors could trigger (retrograde) signaling cascades by the induction of local changes in membrane potential, or through direct interactions with signaling molecules (Sarto-Jackson et al., 2012).

It was recently reported that for perisomatic inhibitory synapses made by parvalbumin-positive basket cells in the visual cortex, activation of GABA_B_ rather than GABA_A_ receptors is involved in regulation bouton dynamics (Fu et al., 2012). This raises the intriguing possibility that perisomatic and dendritic inhibitory synapses have distinct molecular components (Fritschy et al., 2006; Poulopoulos et al., 2009) and display distinct plasticity rules. In general, GABA-dependent regulation of inhibitory axonal dynamics could be a mechanism to adjust the likelihood for the formation of new inhibitory synapses at or near the site of enhanced activity, possibly as part of a local compensation mechanism.

## Concluding remarks

Plasticity of glutamatergic and GABAergic synapses is crucial for balancing excitation and inhibition in our brain. Small and local imbalances can already have profound effects on behavior, reminiscent of diseases such as autism or schizophrenia (Chao et al., 2010; Yizhar et al., 2011; Grillo et al., 2012). Here we report how structural changes in presynaptic inhibitory boutons play into these changes and how they are affected by large scale alterations in neuronal activity. In order to fully understand the complex interplay of excitation and inhibition and how a proper balance is maintained in the healthy brain it will be essential to address the complex mechanisms underlying interactions between inhibitory and excitatory synapses on a synapse-by-synapse basis.

## Author contributions

Anne Schuemann, Tobias Bonhoeffer and Corette J. Wierenga designed the experiments. Anne Schuemann performed the experiments and Anne Schuemann and Corette J. Wierenga analyzed the data. Agnieszka Klawiter contributed raw data for Figures 6B–D. Anne Schuemann, Tobias Bonhoeffer and Corette J. Wierenga wrote the paper.

### Conflict of interest statement

The authors declare that the research was conducted in the absence of any commercial or financial relationships that could be construed as a potential conflict of interest.
